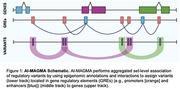# Identification of candidate causal risk genes and pathways in Alzheimer's disease and Lewy body dementia

**DOI:** 10.1002/alz70855_105172

**Published:** 2025-12-24

**Authors:** Danielle M. Picarello, Devynn A. Adams, Tulsi Patel, Brian Fulton‐Howard, Ruth Chia, Betul Comertpay, Alison M. Goate, Alexey Kozlenkov, Stella Dracheva, Bryan J Traynor, Sonja W Scholz, Edoardo Marcora, Alan E. Renton

**Affiliations:** ^1^ Department of Genetics and Genomic Sciences, Icahn School of Medicine at Mount Sinai, New York, NY, USA; ^2^ Ronald M. Loeb Center for Alzheimer's Disease, Icahn School of Medicine at Mount Sinai, New York, NY, USA; ^3^ National Institute on Aging, Bethesda, MD, USA; ^4^ National Institute of Health, Bethesda, MD, USA; ^5^ Neuromuscular Diseases Research Section, Bethesda, MD, USA; ^6^ Nash Family Department of Neuroscience, Icahn School of Medicine at Mount Sinai, New York, NY, USA; ^7^ Department of Psychiatry, Icahn School of Medicine at Mount Sinai, New York, NY, USA; ^8^ Department of Neurology, Johns Hopkins University Medical Center, Baltimore, MD, USA; ^9^ Neurodegenerative Diseases Research Section, National Institute of Neurological Disorders and Stroke, Bethesda, MD, USA

## Abstract

**Background:**

The neurodegenerative disorders Alzheimer's disease (AD) and Lewy body dementia (LBD) share genetic and clinicopathological overlap and may constitute a disease spectrum. Common variant genome‐wide association studies (GWASs) have uncovered AD and LBD etiology. However, common variant GWASs identify loci not genes, hence post‐GWAS functional mapping analyses are needed to elucidate genetic risk mechanisms and drive therapeutic development. AD and LBD genetic risk mechanisms and their overlap are poorly understood. We performed functional mapping, integrating AD and LBD GWAS sumstats with brain cell type‐specific epigenomic datasets and enrichment analysis to nominate candidate causal disease risk genes and pathways.

**Methods:**

Building on multi‐marker analysis of genomic annotation (MAGMA), we developed the novel tool annotation‐ and interaction‐based MAGMA (AI‐MAGMA). AI‐MAGMA integrates GWAS sumstats with chromatin annotation and interaction data to nominate candidate causal risk genes (Figure 1). We used AI‐MAGMA to integrate AD and LBD GWAS sumstats with microglial, neuronal, and oligodendroglial (oligodendrocytes and oligodendrocyte precursor cells) bulk chromatin immunoprecipitation‐sequencing (ChIP‐seq) (annotation) and bulk proximity ligation‐assisted chromatin immunoprecipitation‐seq (PLAC‐seq) (interaction) data to nominate candidate causal genes mediating disease risk in these brain cell types. We performed enrichment analysis with gProfiler to identify disease pathways implicated by these genes.

**Results:**

Integrative functional mapping nominated AD (354 microglial, 203 neuronal, and 144 oligodendroglial) and LBD (40 microglial, 16 neuronal, and 22 oligodendroglial) candidate causal risk genes achieving study‐wide significance (*p*‐value < 0.05/n genes analyzed). AD pathway enrichment highlighted: amyloid, lipid metabolism, endocytosis, phagocytosis, and immunity in microglia; amyloid and neurofibrillary tangles in neurons; and amyloid, lipid metabolism, and blood‐brain barrier in oligodendroglia. LBD pathway enrichment revealed: lipid metabolism, endocytosis, and mitochondria in microglia; synuclein in neurons; and amyloid, lipid metabolism, synapse, and blood‐brain barrier in oligodendroglia.

**Conclusion:**

Functional mapping nominated dozens of AD and LBD candidate causal risk genes. Pathway enrichment implicated microglial lipid metabolism and endocytosis as well as oligodendroglial amyloid, lipid metabolism, and blood‐brain barrier as pathophysiological mechanisms shared across AD and LBD. These analyses also suggested roles for mitochondria and synapses in LBD microglia and oligodendroglia, respectively, enhancing our understanding of LBD genetic risk mechanisms.